# Pseudodidymosis aplasticosebacea: Binary phenomenon of congenital aplasia cutis congenita and nevus sebaceus

**DOI:** 10.1016/j.jdcr.2024.04.014

**Published:** 2024-04-21

**Authors:** Retno Danarti, Rudolf Happle, Ani Rifko, Agnes Rosarina Prita Sari, WenChieh Chen

**Affiliations:** a Faculty of Medicine, Public Health and Nursing, Department of Dermatology and Venereology, Universitas Gadjah Mada, Yogyakarta, Indonesia; b Polyclinic of Dermatology and Venereology, Dr. Sardjito General Hospital, Yogyakarta, Indonesia; c Department of Dermatology, Medical Center-University of Freiburg, Freiburg im Breisgau, Germany; d Department of Dermatology and Allergy, Technische Universität München, Munich, Germany

**Keywords:** aplasia cutis congenita, binary genodermatosis, nevus sebaceus, pseudodidymosis aplasticosebacea

## Introduction

The concurrence of aplasia cutis congenita (ACC), nevus sebaceus (NS) , and ophthalmic abnormality, including limbal dermoid, has been reported in the literature.^1^, ^2^, ^3^ ACC is a congenital absence of a portion of skin in a localized or widespread area. It commonly affects the scalp but can also affect any other body part.^4^

Sebaceous glands are typically undeveloped during childhood. The presence of less differentiated structures resembling embryonic hair follicles is a common finding in early childhood.^5^ The excessive growth of sebaceous glands characterizes NS, which is a mosaic RASopathy resulting from postzygotic mutations involving either *HRAS* or *KRAS* genes.^6^

In a review of 16 cases with concurrence of ACC and NS, including their own one, Happle and König proposed the term didymosis aplasticosebacea, which may explain the coexistence of these 2 different congenital skin disorders.^1^ In 2023, Happle and Toriello stated that nevi appearing in a binary manner are primarily caused by a single mutation in a pluripotent progenitor cell, leading to multiple manifestations in various tissues. The idea of nonallelic didymosis can no longer be supported in molecular studies, which is why the term "pseudodidymosis" has been employed to replace didymosis to describe this group of binary disorders.^7^

We present a case of NS syndrome associated with ACC in the form of pseudodidymosis aplasticosebacea, accompanied by neurological and ocular anomalies.

## Case report

A five-month-old boy presented to us with a congenital deformity of the left scalp area and eye. His mother exhibited multiple hyperpigmented macules on the face, body, and extremities, which were also found in mother’s father, brothers, and sisters. We diagnosed these clinical picture as multiple lentiginosis. His parents are not consanguineous. There is no history of seizures in the patient. During pregnancy, the mother was healthy, did not have any complaints, denied any history of illnesses, and did not consume nonsteroidal anti-inflammatory drugs or teratogenic drugs such as methimazole.

**Fig 1:**
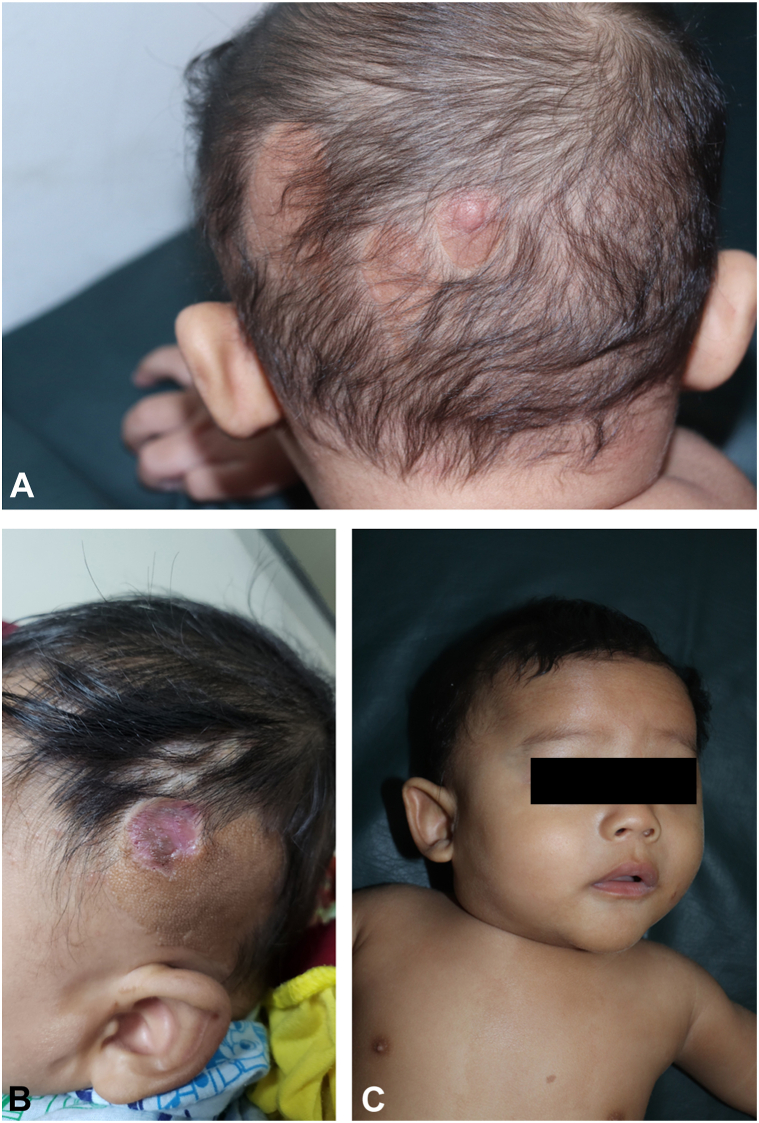
**A,** Multiple *yellowish* plaques with velvety surface in the occipital region, (**B**) skin defect in the left temporal area (age 7 month old), (**C**) Limbal dermoid of the left eye.

**Fig 2:**
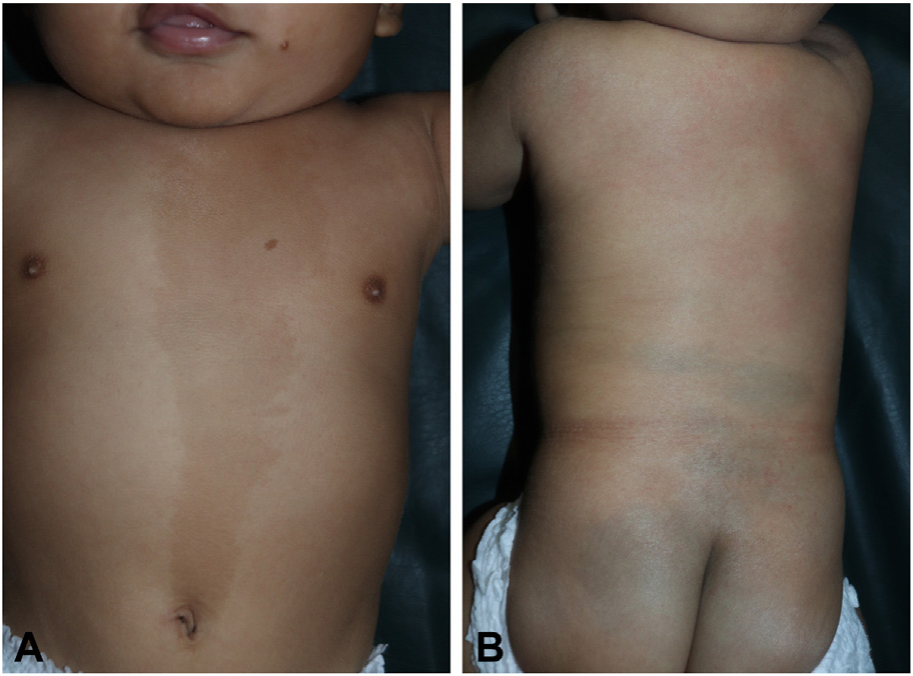
**A,** Flag-like café-au-lait patch on the chest with sharp *mid-line* separation and hyperpigmented macules, (**B**) Dermal melanocytosis on the lower back and buttocks.

**Fig 3:**
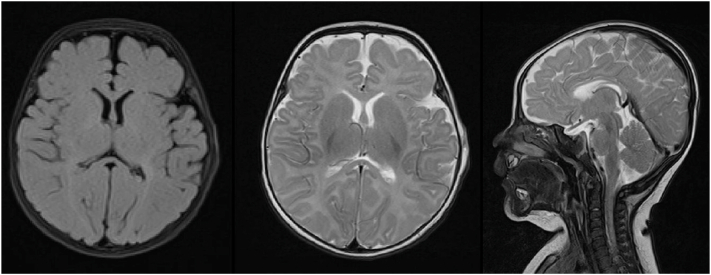
Magnetic resonance imaging (MRI) showed bilateral dilation of the frontotemporal subarachnoid space and bilateral hyperintense lesions of the putamen and thalamus.

**Fig 4:**
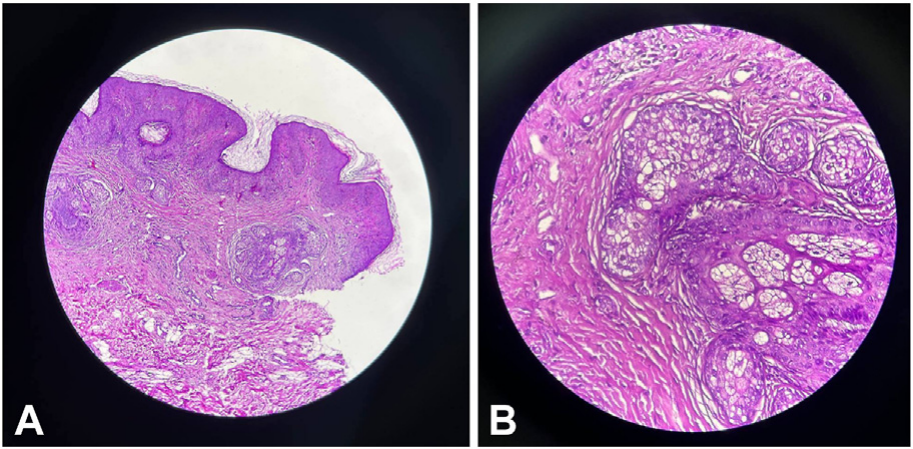
**A,** Basket weave type orthokeratosis of epidermis and irregular acanthosis, upper dermis with immature pilosebaceous units, apocrine glands, proliferation and dilatation of blood vessels, and infiltration of patchy inflammatory cells, with light density, consisting of lymphocytes, histiocytes, and neutrophils, especially in the papillary dermis and peri-adnexal (×100). **B,** ×400.

At birth, flat, hairless, yellow-brown skin lesions arranged in a patchy pattern were noted in the left temporo-occipital area of the scalp. A circumscribed, well-demarcated hairless patch with an atrophic surface, consistent with ACC, was directly adjacent to, and partly intermingled with, the elevated yellowish lesions (Fig 1, *A* and *B*). A limbal dermoid of the left eye, diagnosed by ophthalmologist, had resulted in partial corneal opacity (Fig 1, *C*) In addition, he exhibited a café-au-lait patch on the left side of his chest, being superimposed by hyperpigmented maculae (Fig 2, *A*) and segmental dermal melanocytosis on his lower back and buttocks (Fig 2, *B*). Magnetic resonance imaging revealed bilateral frontotemporal subarachnoid space enlargement and bilateral hyperintense lesions in the putamen and thalamus (Fig 3). Histopathological examination revealed basketweave type orthokeratosis of the epidermis and irregular acanthosis. The upper dermis with immature pilosebaceous units, apocrine glands, proliferation and dilation of blood vessels, and infiltration of patchy inflammatory cells, with light density, consisting of lymphocytes, histiocytes, and neutrophils, particularly in the papillary dermis and periadnexal, was noted. The lower dermis showed irregularly arranged collagen suggestive of NS (Fig 4, *A* and *B*). NS syndrome associated with ACC in the form of pseudodidymosis aplasticosebacea was diagnosed.

## Discussion

The incidence of ACC is estimated to be between 0.5 and 1 in 10,000 newborns.^8^ The cause is heterogeneous and the clinical appearance varies, ranging from erosion, ulceration, or ovoid defect covered by a membrane.^9^^,^^10^ In 70% of patients, the disorder occurs as a solitary defect on the scalp.^1^ It could also occur in association with other anomalies and is sometimes noted in close proximity to a NS. The diagnosis of ACC is based on physical examination, and biopsy is normally not required.^11^

NS is a benign congenital skin lesion that typically affects the scalp and face. It is a hamartoma composed of epidermal, sebaceous, and apocrine components. Hairless, yellow-orange plaques of variable size and form characterize the clinical appearance.^5^ The sebaceous component in our patient is most likely identical to that in the sebaceous nevus syndrome, which includes sebaceous nevus and cerebral, ocular, or skeletal abnormalities.^12^

Our patient has left eye limbal dermoid. Eleven patients reported with pseudodidymosis aplasticosebacea had ocular defects,^1^, ^2^, ^3^ and 8 cases presented with associated central nervous system anomalies.^1^^,^^3^ The bilateral dilation of the frontotemporal subarachnoid space in our case, with bilateral hyperintense lesions of the putamen and thalamus had also been reported before.^13^ In their review of co-occurrence of ACC and NS Happle and König reported their 1-year-old boy patient who had flag-like hyperpigmentation of the trunk.^1^ Our patient has a café au lait patch on his chest superimposed with hyperpigmented maculae and diffuse dermal melanocytosis on his lower back and buttocks, which has not been reported before. In conclusion NS syndrome associated with ACC in the form of pseudodidymosis aplasticosebacea, accompanied by neurological and ocular anomalies is sometimes reported in the literature. It is probable that this isn't an arbitrary coincidence and has to do with a particular, as yet undiscovered biochemical signaling pathway connecting the sebaceous cells to the surrounding tissue.

## Conflicts of interest

None disclosed.
